# Commentary: “Estrogenic and Anti-Androgenic Endocrine Disrupting Chemicals and Their Impact on the Male Reproductive System”

**DOI:** 10.3389/fpubh.2015.00165

**Published:** 2015-06-22

**Authors:** Francisco José Roma Paumgartten

**Affiliations:** ^1^National School of Public Health, Oswaldo Cruz Foundation (FIOCRUZ), Rio de Janeiro, Brazil

**Keywords:** endocrine disrupting chemicals, bisphenol A, semen quality, male reproductive system, review, epidemiology, reproductive health, environmental health

During the last two decades or so, endocrine-disrupting chemicals (EDCs) and their effects on human health have become one of the most researched and controversial topics in toxicology. There are a number of reviews on the health consequences of exposure to EDCs including a comprehensive report by the World Health Organization and the United Nations Environment Programme (1). Recently, De Falco et al. (2) addressed the impact of EDCs on male reproductive system, with special reference to the effects of bisphenol A (BPA), alkylphenols, and phthalates. Jeng (3) also reviewed the epidemiological data on the adverse effects of EDCs on male reproduction and experimental studies that could shed light on mechanisms (disruption of steroidogenesis, oxidative stress, and epigenetic changes) through which EDCs could impair male reproductive health. Both articles are essentially narrative reviews of the abundant and highly controversial literature on the health consequences of exposures to EDCs.

A key feature that distinguishes a narrative review from a systematic review is that the former review does not include a comprehensive and meticulous search of all potentially relevant articles on specified sources, and does not use explicit and reproducible criteria to selected articles for review (4). Compared to systematic reviews, narrative reviews of the literature are more likely to error and bias in the selection of relevant studies (4, 5). Moreover, if research designs, methods, and study characteristics do not undergo a critical appraisal, summary, and conclusions of literature reviews are even more prone to bias.

De Falco et al. were unable to convey to readers an unbiased review of the empirical evidence suggesting that environmental exposures to EDCs might affect male reproduction. The authors, for instance, did not disclose the conflicting evidence on the enlargement of prostate after developmental exposure to BPA. In the mid-1990s, a set of studies by vom Saal and coworkers showed that prenatal exposure to β-estradiol (EST), diethylstilbestrol (DES), or BPA led to enlarged ventral prostate in adult mice (6, 7). The observation that enlargement of prostate resulted from prenatal exposures to low doses of estrogenic compounds (e.g., supra-physiological levels of EST), and exhibited non-monotonic dose–response relationships, fueled considerable debate over the adverse health consequences of environmental exposure to EDCs. Several studies, however, failed to reproduce these findings not only with BPA but also with EST and DES (8–10). Although reproducibility is one hallmark of experimental sciences, the foregoing discrepancy between studies by different authors has remained unexplained (11).

Furthermore, authors’ statements that “…over 50 years, the global average sperm count dropped by half…” and that “studies of the last decade strongly support that male reproductive health has been deteriorating…” were unaccompanied by any reference to the conflicting evidence on this matter (2). The widespread notion that semen quality has decreased over the past decades stands on some retrospective studies [Ref. (12, 13), and others]. Nonetheless, results from a number of other studies (not cited by the authors) are inconsistent with this hypothesis. Most studies showing downward trends in sperm counts included samples coming from different populations and places that do not necessarily allow a valid comparison over time. For instance, a re-analysis of US data used by Carlsen et al. (12) found no decline in sperm counts when data from New York were excluded from the regression analysis (14). Therefore, apparent time trend toward lower concentrations reported by Carlsen et al. (12) resulted, in fact, from geographic variations in sperm counts (14, 15). Moreover, a longitudinal study of sperm concentrations for Danish military draftees (5000 men), collected annually for 15 years (1996–2010), found no indication that semen quality has changed during the monitoring period (16). Although several studies precipitated by reports on “downward temporal trends in sperm counts” refuted its existence, the “sperm crisis” notion is still a highly controversial issue in the literature (17–20). Temporal trends to increasing birth prevalence of male reproductive tract defects such hypospadias and cryptorchidism described by some authors are far from being a consistent finding among studies (21, 22).

The “endocrine disruptor hypothesis,” a landmark of which was the Wingspread Conference Statement in 1990s (23), fits like a glove to the beliefs of the public that pesticides and other manmade chemicals in the environment are undermining human health and fertility. Two seminal books by Rachel Carlson (Silent Spring, 1962) and Theo Colborn (Our Stolen Future, 1996) boosted considerably these concerns on the deleterious effects of environmental chemicals on human fertility and health. Notwithstanding the fact that ED hypothesis is instigating, the notion that “male reproductive health has been deteriorating,” as asserted by De Falco et al. (2) and others, lacks an unequivocal demonstration by soundly designed epidemiology studies. It is of note that, even if a temporal trend toward a worse male reproductive health had been demonstrated consistently, it would still be missing to prove that there is a causal link between EDCs, identified as such in experimental tests, and the incidence of the adverse health outcome in the human population. A step forward to identifying relevant research gaps and to unveiling the real impact of EDCs on male fertility and reproductive health would be to conduct less and less narrative and potentially biased reviews and more and more good quality systematic reviews of the literature on the topic. Finally, we highlight that a critical appraisal of the quality of original studies is required for both a good quality narrative and a good quality systematic review. If unbiased, good quality narrative reviews can also be helpful. Systematic reviews, however, are a more reliable approach to avoid bias in the selection of studies.

## Conflict of Interest Statement

The author declares that the research was conducted in the absence of any commercial or financial relationships that could be construed as a potential conflict of interest.

## References

[1] World Health Organization. State of the science of endocrine disrupting chemicals – 2012. In: Bergman A Heindel JJ Jobling S Kidd KA Zoeller RT, editors. Geneva: WHO Press (2013). Available from: http://www.who.int/ceh/publications/endocrine/en/

[2] De Falco M Forte M Laforgia V Estrogenic and anti-androgenic endocrine disrupting chemicals and their impact on the male reproductive system. Front Environ Sci (2015) 3:3 10.3389/fenvs.2015.00003 PMC4476199 26157793

[3] Jeng HA. Exposure to endocrine disrupting chemicals and male reproductive health. Front Public Health (2014) 2:55. 10.3389/fpubh.2014.00055 24926476 PMC4046332

[4] Cook DJ Mulrow CD Haynes RB. Systematic reviews: synthesis of best evidence for clinical decisions. Ann Intern Med (1997) 126:376–80. 10.7326/0003-4819-126-5-199703010-00006 9054282

[5] Mulrow CD Rationale for systematic reviews. BMJ (1994) 309:597–9. 10.1136/bmj.309.6954.597 8086953 PMC2541393

[6] Nagel SC vom Saal FS Thayer KA Dhar MG Boechler M Welshons WV. Relative binding affinity-serum modified access (RBA-SMA) assay predicts the relative in vivo bioactivity of the xenoestrogens bisphenol A and octylphenol. Environ Health Perspect (1997) 105:70–6. 10.1289/ehp.9710570 9074884 PMC1469837

[7] vom Saal FS Timms BG Montano MM Palanza P Thayer KA Nagel SC Prostate enlargement in mice due to fetal exposure to low doses of estradiol or diethylstilbestrol and opposite effects at high doses. Proc Natl Acad Sci U S A (1997) 94:2056–61. 10.1073/pnas.94.5.2056 9050904 PMC20042

[8] Ashby J Tinwell H Haseman J. Lack of effects for low dose levels of bisphenol A and diethylstilbestrol on the prostate gland of CF1 mice exposed in utero. Regul Toxicol Pharmacol (1999) 30:156–66. 10.1006/rtph.1999.1317 10536110

[9] Cagen SZ Waechter JM Jr Dimond SS Breslin WJ Butala JH Jekat FW Normal reproductive organ development in CF-1 mice following prenatal exposure to bisphenol A. Toxicol Sci (1999) 50:36–44. 10.1093/toxsci/50.1.36 10445751

[10] Tyl RW Myers CB Marr MC Sloan CS Castillo NP Veselica MM Two-generation reproductive toxicity study of dietary bisphenol A in CD-1 (Swiss) mice. Toxicol Sci (2008) 104:362–84. 10.1093/toxsci/kfn084 18445619

[11] Paumgartten FJR De-Oliveira ACX The bisphenol A toxicological paradox: the more we learn the less we know for sure. Environ Skeptics Critics (2014) 3:65–82.

[12] Carlsen E Giwercman A Keiding N Skakkebaek NE. Evidence for decreasing quality of semen during past 50 years. BMJ (1992) 305:609–13. 10.1136/bmj.305.6854.609 1393072 PMC1883354

[13] Auger J Kunstmann JM Czyglik F Jouannet P. Decline in semen quality among fertile men in Paris during the past 20 years. N Engl J Med (1995) 332:281–5. 10.1056/NEJM199502023320501 7816062

[14] Saidi JA Chang DT Goluboff ET Bagiella E Olsen G Fisch H. Declining sperm counts in the United States? A critical review. J Urol (1999) 161:460–2. 10.1016/S0022-5347(01)61923-2 9915426

[15] Fisch H. Declining worldwide sperm counts: disproving a myth. Urol Clin North Am (2008) 35:137–46. 10.1016/j.ucl.2008.01.001 18423235

[16] Bonde JP Ramlau-Hansen CH Olsen J Trends in sperm counts: the saga continues. Epidemiology (2011) 22:617–9. 10.1097/EDE.0b013e318223442c 21629118

[17] Jørgensen N Vierula M Jacobsen R Pukkala E Perheentupa A Virtanen HE Recent adverse trends in semen quality and testis cancer incidence among Finnish men. Int J Androl (2011) 34:e37–48. 10.1111/j.1365-2605.2010.01133.x 21366607 PMC3170483

[18] Bonde JP Jensen MS Ramlau-Hansen CH Toft GV Thulstrup AM Olsen J Trends in sperm count in Finnish men. Int J Androl (2012) 35:626 10.1111/j.1365-2605.2011.01214.x 21875429

[19] Sharpe RM Sperm counts and fertility in men: a rocky road ahead. Science & society series on sex and science. EMBO Rep (2012) 13:398–403. 10.1038/embor.2012.50 22491033 PMC3343360

[20] Nieschlag E Lerchl A Sperm crisis: what crisis? Asian J Androl (2013) 15:184–6. 10.1038/aja.2012.90 23001441 PMC3739166

[21] Toledano MB Hansell AL Jarup L Quinn M Jick S Elliott P. Temporal trends in orchidopexy, Great Britain, 1992-1998. Environ Health Perspect (2003) 111(1):129–32. 10.1289/ehp.5446 12515691 PMC1241317

[22] Abdullah NA Pearce MS Parker L Wilkinson JR Jaffray B McNally RJ. Birth prevalence of cryptorchidism and hypospadias in northern England, 1993-2000. Arch Dis Child (2007) 92:576–9. 10.1136/adc.2006.102913 17142312 PMC2083772

[23] Krimsky S Environmental endocrine hypothesis and public policy. In: Kroll-Smith S Brown P Gunter VJ, editors. Illness and the Environment. New York, NY: University Press (2000). p. 95–107.

